# 5S rRNA: Structure and Function from Head to Toe

**Published:** 2005-06

**Authors:** Olga A. Dontsova, Jonathan D. Dinman

**Affiliations:** 1 *Department of Chemistry, Moscow State University, Building A, Room 608, Vorobievy Gory, Moscow, Russia;*; 2 *Department of Cell Biology and Molecular Genetics, Microbiology Building Room 2135, University of Maryland, College Park, USA*

**Keywords:** Ribosome, rRNA, 5S, translation, fidelity, frameshifting, virus

## Abstract

5S rRNA is uniquely positioned so as to link together all of the functional centers of the ribosome. Previous studies have supported the hypothesis that 5S rRNA acts as a physical transducer of information, facilitating communication between the different functional centers and coordinating of the multiple events catalyzed by the ribosome. Here, we present a synthesis of both structural and genetic information to construct a more detailed picture of how 5S rRNA may act to transmit and coordinate all of the functional centers of the ribosome.

## INTRODUCTION

The availability of detailed structural information, biochemical assays, and molecular genetic systems make the ribosome a robust model for studying how the structures of complex molecules dictate function at the molecular level. This megadalton complex is composed of multiple proteins and RNAs in which the activities of numerous functional centers are coordinated to synthesize proteins with great accuracy. In simple terms the ribosome coordinates the activities of peptide synthesis by proceeding through a series of at least 9 functional centers in at least 7 discrete unidirectional steps (1). Analyses of static X-ray crystal structures have been useful in assigning the positions of the functional centers at the atomic level, revealing for example that the catalytic activity of the ribosome is mediated by RNA, and identifying the binding sites for antibiotics (1-4). Cryo-EM studies provide complementary information, showing dynamic views of intra-ribosome movements through many of the different phases of the translation cycle (5).

5S rRNA is the smallest RNA component of the ribosome, and its secondary structure has been determined for many living organisms (6). Although the tertiary structure of 5S rRNA has been obtained for the uncomplexed molecule (7, 8), its isolated domains (9-11), and as a part of a ribosomal complex (12-15), the precise function of 5S rRNA in protein synthesis is not fully understood. Biochemical studies with E. coli ribosomes led to the hypothesis that 5S rRNA acts as a physical transducer of information, facilitating communication between the different functional centers and coordinating the multiple events catalyzed by the ribosome (16, 17), and this view was further supported by a later study in yeast ribosomes (18). More recently, we have also characterized the effects of this 5S rRNA saturation library on programmed -1 and +1 ribosomal frameshifting (PRF) (19). Exploitation of the 5S rRNA mutants combined with phenotypic and structural analyses of the pure mutants is helping to provide a clearer understanding of how 5S rRNA may act to coordinate the multiple functional centers of the ribosome.

### The "head" of the 5S rRNA is in the upper part of central protuberance

**Figure 1:**
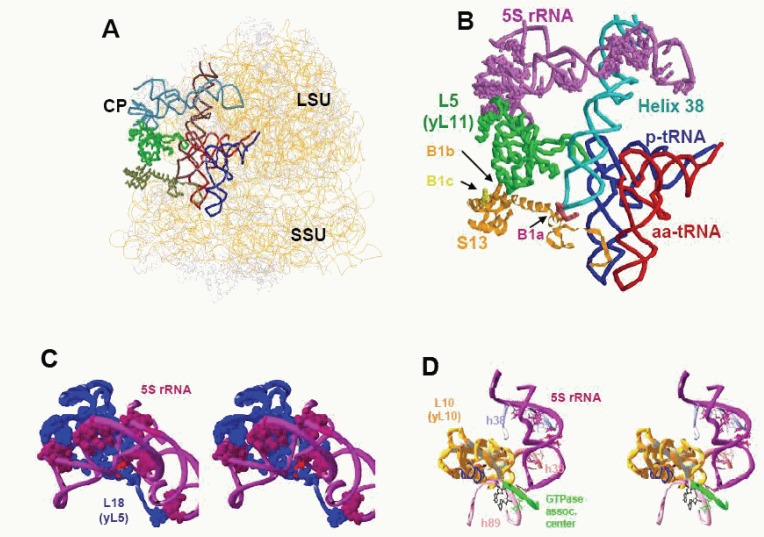
Interactions between 5S rRNA and other regions of the ribosomes. (**A**), Lateral view of the E. coli ribosome adapted from (42). 5S rRNA is colored light blue. Large subunit (LSU), small subunit (SSU), and central protuberance (CP) are labelled; (**B**), Closeup of interactions that link 5S rRNA to intersubunit bridges, the decoding center and the A- and P-site tRNAs. 5S rRNA alleles that affect translational fidelity are shown as purple spheres (18, 19); (**C**), Interaction between 5S rRNA and ribosomal protein L18 (yeast L5). Alleles of yeast 5S rRNA that affect translational fidelity are shown as purple spheres (18, 19). The T28A allele of yeast L5 (34) is indicated as a red sphere; (**D**), 5S rRNA alleles in the "toe" region that affect translational fidelity are indicated.

5S rRNA is a major component of the central protuberance of the large ribosomal subunit (Fig 1A). The molecule itself does not directly contact either the P- or A-site bound tRNAs, nor is it a component of the peptidyltransferase, decoding, or elongation factor binding centers. However, it is uniquely positioned so as to be able to connect all of these components with one another. The upper part of the central protuberance is composed of the 5S rRNA helices I, II, and III, connected by the A, B, and C loops respectively. It is sandwiched between ribosomal proteins L5 (bacterial L18) on the solvent side of the LSU and L11 (bacterial L5) together with helix 85 on the interior face (Fig. 1B, 1C). Protein L11 (bacterial L5) forms the conserved B1b/c bridge (20) with the small subunit ribosomal protein S15 (bacterial S13, Fig. 1B) (14). Importantly, the C-terminal tail of S13 is located between the anticodon arms of the A- and P-site tRNAs (21, 22), thus establishing a link between 5S rRNA and the decoding center on the small subunit (Fig. 1B).

The intersubunit bridges, formed by central protuberance of large ribosomal subunit could be a part of allosteric signal transmission conduit between the decoding center on the small ribosomal subunit and the functional centers on the large subunit. The upper area of the central protuberance is relatively mobile (23), although conformation changes could not be detected by structure probing (24). In both bacteria (23) and yeast (25), the intersubunit bridge B1b undergoes rearrangement upon binding of EF-G (eEF-2). In E. coli the B1b bridge is formed between protein L5 complexed 5S rRNA and the small subunit protein S13, and the contact sites between L5 and S13 are changed during the course of translocation. In yeast ribosomes, the actual partner of L11 (the homolog of bacterial L5) is changed upon eEF-2 binding: in the absence of eEF-2, L11 contacts S18 (the bacterial S13 homolog) (14), but after eEF-2 binding the partner of L11 changes to S15, the homolog of bacterial S19 (26).

In addition to its contact with small subunit, L11 (bacterial L5) interacts with P-site bound tRNA (1, 2, 5, 27, 28). Biochemical and structural studies show that the status of the large subunit P-site is monitored to regulate the enzymatic activities of the elongation factors (29, 30), and we have shown that post-peptidyltransfer ribosomes do not slip in the -1 direction (reviewed in 31). A synthesis of these observations suggests that the status of P-site tRNA body is monitored by L5 (yeast L11), i.e. L5 helps to determine whether this tRNA is in the P/P or P/E hybrid state. A large number of the mutations in the “head” of 5S rRNA were also shown to inhibit -1 frameshifting (Fig. 1B) (19). According to our current models, -1 frameshifting happens during or immediately after the accommodation step, and clearly before translocation (31, 32). Stabilization of the post-translocational state would interfere with -1 frame-shifting, while at the same time increasing the probability of the +1 frameshift. Additionally, decreased translational fidelity in the form of nonsense codon suppression tend to cluster in this region (18).

The “head” of 5S rRNA also makes extensive contacts with yeast ribosomal protein L5 (bacterial L18), which correspond to the sites in 5S rRNA that produce translational fidelity phenotypes upon mutation (Fig. 1C). The mutation of threonine 28 of L5 to alanine (red sphere in Fig. 1C) is of particular interest, as was shown it promoted temperature sensitivity, ribosome biogenesis defects (33), sparsomycin resistance, and increased -1 and +1 PRF as a consequence of decreased ribosome affinity for peptidyl-tRNA (34). This threonine is at the center of highly coordinated interaction between 5S rRNA and L5, and close inspection of the large subunit crystal structures reveals that the hydroxyl group of threonine forms a hydrogen bond with the phosphate backbone of 5S rRNA between G7 and G8. These findings suggest that disruption of contacts between L5 and 5S rRNA negatively impact the ability of this region of ribosome to interact with peptidyl-tRNA.

### The interaction between the “middle” of 5S rRNA and the A-site finger

The "middle" part of 5S rRNA, composed of helices IV, V, and loop E, connects the upper part of central protuberance with loop D of 5S rRNA. This loop is directly involved in structural organization of 25S rRNA domain II, connecting the peptidyltransferase region with the "GT-Pase associated center" The "middle" of 5S rRNA forms a contact with the highly conserved helix 38 of 25S rRNA (the "A-site finger”, which forms a prominent extension that juts out of the intersubunit face of the large subunit toward the small subunit (Figs. 1A and 1B). In bacteria it takes part in formation of the B1a bridge with small ribosomal subunit proteins S13 (20) and S19 (23). In yeast, helix 38 also participates in formation of the B1a bridge where the small subunit partner is S15 (bacterial S19). Like B1b, the B1a also undergoes conformational changes upon binding of EF-G (eEF-2). In bacteria, the B1a bridge is completely disrupted upon binding of EF-G (23), whereas binding of eEF-2 causes the A-site finger to change its interaction partner to the helix 33 of 18S rRNA in yeast ribosomes (26). Although the B1a and B1b bridges are formed by different components of the large ribosomal subunit, both undergo the most prominent changes as compared to other intersubunit contacts, and their large subunit partners, i.e. L5 (yeast L11) and the A-site finger, are both connected to 5S rRNA.

While the B1b bridge is located above the P-site bound tRNA, cryo-EM (35, 36) and crosslinking studies (27, 37) show that the B1a bridge contacts the A-site tRNA. Thus, this interaction serves to link 5S rRNA to the A-site of the decoding center. The phenotypes of the 5S rRNA alleles located in the A-site finger interacting "middle" part of the molecule were different from those in the "head" Specifically, whereas all of the mutants in the "middle" of 5S rRNA alleles acted to inhibit +1 PRF (19), their effects on -1 PRF were allele-specific, and none affected nonsense suppression (18). Since +1 PRF occurs in the post-translocation state, it is likely that 5S rRNA mutations localized in the “middle” region affect translocation.

### The "toe" of the 5S rRNA: interplay between the peptidyltransferase center and elongation factor binding site

The 5S rRNA "toe" composed of the loop D and helix IV, is located along the putative signaling pathway between the peptidyltransferase center and the elongation factor binding site (Fig. 1D). Cross-linking studies revealed its proximity to helix 89, the component of domain V in the large subunit rRNA that is located near the peptidyltransferase region (38). The same 5S rRNA segment formed cross-links with helices 39 and 42 in domain II, proximal to “GTPase associated center” (16, 39). These contacts were later confirmed by X-ray structure analysis (for review see 28). The hypothesis that 5S rRNA may be involved in allosteric signal transmission between the peptidyltransferase and the "GTPase associated center" was suggested prior to the availability of the atomic resolution structures (17). This idea was later supported by mutagenesis of 5S rRNA itself (18), and of one of its 23S partners (40).

Loop D of 5S rRNA binds several loops of the large subunit rRNA. Its role as a clamp to stabilize interactions between domains II and V was previously proposed both because it was required for peptidyltransferase activity, and because the absence of 5S rRNA could be partially compensated by addition of antibiotics that served to link these two domains together (41). Intriguingly, loop D protrudes into a pocket formed by the helix 42 region connected to the "GTPase associated center" This may provide a contact point through which 5S rRNA could mediate the allosteric transmission of information from the decoding center via the B1a/B1b bridges to the elongation factor binding site. By this process the “head” of 5S rRNA may be able to communicate with the “toe” affecting the position of the “GTPase associated center” and, as suggested by chemical protection studies, possibly the sarcin-ricin loop (19).

Mutations that map to the “toe” of 5S rRNA affected both +1 and -1 PRF (19), but only mutations at position 91 were able to suppress nonsense mutations (18). Specifically, +1 frameshifting tended to be inhibited while -1 PRF was stimulated (19). Since +1 PRF takes place in the post-translocation state, while -1 is thought to happen during accommodation, it is possible that “toe” mutations interfere with eEF-2 (EF-G) activity while stimulating eEF-1 (EF-Tu). Curiously, the effects of the mutations of the “toe” of the 5S rRNA are directly opposite those in the “head”.

Loop D is at the tip of the “toe” of 5S rRNA, where it directly contacts helix 42, which is connected to the large subunit’s “GTPase associated center” (Fig. 1D). It is possible that 5S rRNA influences the positioning/function of this center through this interaction. The 5S rRNA “toe” also contacts helices 39 and 89. Helix 89 is located parallel to helix 91, the tips or their loops are connected by a basepair. The opposite site of the loop-end of helix 91 also interacts with the sarcin-ricin loop, the second elongation factor binding site. Thus, 5S rRNA may also influence the structure of both elongation factor binding sites.

**Figure 2:**
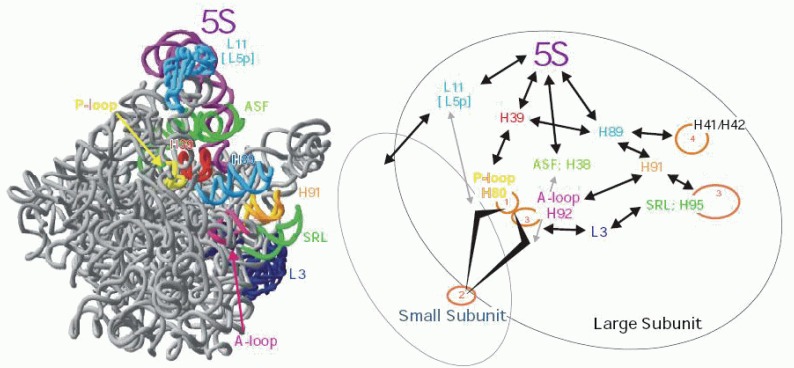
Model of how 5S rRNA may help mediate the allosteric transmission of information among the different functional centers of the ribosome. Left: view of the large subunit from the intersubunit face. In addition to helices and loops, the A-site finger (ASF), sarcin/ricin loop (SRL0, and ribosomal protein L3 are labelled. Right: Diagrammatic representation of the allosteric transmission pathways in the ribosome. 5S RNA relays information regarding both the status of the P-site (grey arrow from L11 to the P-loop, circled 1), and that of the B1b intersubunit bridge (black arrow to S15/S18), which communicates with the decoding center on the small subunit (circled 2). The “middle” also communicates with the P-site through helix 39, and through helix 38 to both the A-site (circled 3), and with the B1a bridge, providing feedback to the decoding center regarding the status of the A-site. The “toe” of 5S rRNA mediates the transmission of information to the elongation factor binding elements, i.e. the sarcin-ricin loop (SRL;H95 circled 3), and the “GTPase associated center” (helices 41/41, circled 4). This information can potentially feed back to the peptidyltransferase center as well through helices 91, 92 and ribosomal protein L3.

Figure 2 presents a model of how 5S rRNA may be involved in mediating the allosteric transmission of information among the different functional centers of the ribosome. The "head" of 5S rRNA relays information regarding both the status of the P-site (grey arrow from L11 to the P-loop), and that of the B1b intersubunit bridge, which communicates with the decoding center on the small subunit (black arrow to S15 to circled 2). Similarly, the "middle" communicates with the A-site (circled 3), and with the B1a bridge which provides feedback to the decoding center regarding the status of the A-site. The "toe" of 5S rRNA mediates the transmission of information to the elongation factor binding elements, i.e. the sarcin-ricin loop (circled 3) and the "GTPase associated center" (circled 4), and potentially to back to the peptidyltransferase center as well through helices 91, 92 and ribosomal protein L3. As such all of the functional centers of the ribosome can be linked by 5S rRNA.
